# Limited capacity of tree growth to mitigate the global greenhouse effect under predicted warming

**DOI:** 10.1038/s41467-019-10174-4

**Published:** 2019-05-15

**Authors:** Ulf Büntgen, Paul J. Krusic, Alma Piermattei, David A. Coomes, Jan Esper, Vladimir S. Myglan, Alexander V. Kirdyanov, J. Julio Camarero, Alan Crivellaro, Christian Körner

**Affiliations:** 1 Department of Geography, University of Cambridge, Cambridge, CB2 3EN UK; 2 Swiss Federal Research Institute (WSL), 8903 Birmensdorf, Switzerland; 3 Global Change Research Centre and Masaryk University, 613 00 Brno, Czech Republic; 4 Department of Physical Geography, Stockholm University, 10691 Stockholm, Sweden; 5 Department of Plant Sciences, University of Cambridge, Cambridge, CB2 3EA UK; 6 Departmemt of Geography, Johannes Gutenberg University, 55099 Mainz, Germany; 7 Institute of Humanities, Siberian Federal University, 660041 Krasnoyarsk, Russia; 8 Sukachev Institute of Forest SB RAS, 660036 Krasnoyarsk, Russia; 9 Institute of Ecology and Geography, Siberian Federal University, 660041 Krasnoyarsk, Russia; 10 Instituto Pirenaico de Ecología (IPE-CSIC), 50059 Zaragoza, Spain; 11 Institute of Botany, University of Basel, 4056 Basel, Switzerland

**Keywords:** Ecology, Climate-change ecology, Ecosystem ecology, Ecosystem services, Forest ecology

## Abstract

It is generally accepted that animal heartbeat and lifespan are often inversely correlated, however, the relationship between productivity and longevity has not yet been described for trees growing under industrial and pre-industrial climates. Using 1768 annually resolved and absolutely dated ring width measurement series from living and dead conifers that grew in undisturbed, high-elevation sites in the Spanish Pyrenees and the Russian Altai over the past 2000 years, we test the hypothesis of grow fast—die young. We find maximum tree ages are significantly correlated with slow juvenile growth rates. We conclude, the interdependence between higher stem productivity, faster tree turnover, and shorter carbon residence time, reduces the capacity of forest ecosystems to store carbon under a climate warming-induced stimulation of tree growth at policy-relevant timescales.

## Abstract (web summary)

Fertilization under greenhouse warming conditions is expected to accelerate tree growth and potentially increase the biological storage of CO_2_. Here the authors analyse ring width measurements from 1768 conifers from the Spanish and Russian mountains and demonstrate that longevity requires slow growth rates at least in mountainous regions.

## Introduction

Despite a wide range of recent advancements in tree-ring research^1^, including contributions to (paleo)climatology, ecology, plant physiology and wood anatomy, it is still not clear if tree longevity depends on slow growth rates, and whether or not this relationship is species-specific, genetic and/or environmentally controlled. Our lack of understanding has an important bearing on the current debate about carbon sequestration^2^, carbon residence time^3^, and climate change mitigation^4–7^. This knowledge gap is disconcerting as faster tree growth under future climate change is expected to lead to higher forest carbon stocks, thereby contributing to the mitigation of the anthropogenic greenhouse effect via the biological uptake of carbon dioxide (CO_2_). The assumption that a climate warming-induced increase of tree growth translates into large-scale carbon sequestration is a paradigm that has far-reaching political, ecological and economic consequences^8,9^. The concept of negative emission, vis-a-vis secondary forests, has generated much governmental and institutional action^8,10^; the Bonn Challenge being one example^11^, already causing a multitude of societal and environmental implications^12^.

The putative tradeoff between the rate of tree growth and achieved tree lifespan is complicated by complex interactions between the composition and density of forest stands and the possibility that trees can switch their growth strategy once they reach a certain size^13^. Accelerated growth rates of juvenile trees in dense forest stands permit individual trees to escape from becoming victims of competitive exclusion, which potentially turns into increased longevity, both within monocultures and mixed populations^14,15^. In more open forests, such as those of the upper alpine and northern boreal treeline ecotones, a distinction between interspecific and intraspecific effects on tree longevity is needed. Though it is generally accepted that fast growing, pioneer tree species exhibit overall lower wood density and a shorter life expectancy^16^, which jointly translates into a limited capacity for carbon sequestration, it is still unknown if later successional species growing under cold and temperate climates could live longer if they grew slower during their late adolescence and early adult life. Since this seems to be the case for many humid tropical taxa^17,18^, tree mortality rates are often positively correlated with forest net primary productivity^18^, whereas our understanding of size-specific tree mortality patterns is challenged by a lack of suitable data^19^.

Trees that grow fast beyond their juvenile seedling-sapling stage, commonly exhibit an accelerated life cycle^20^, whereas slower growing individuals get older and taller^21–23^. This observation resembles the rate of life concept in animal sciences^24^, in which high metabolic rates are negatively correlated with longevity. Such a diversity of life histories affects the architecture, age structure, and biomass turnover rates of forest communities^25^, and thus, to a large degree, the terrestrial carbon stock^4,9^. On a global scale, the short but fast life of trees is associated with reduced carbon reservoirs (short carbon residence time)^20^. For example, the highly productive southern and western Amazonian forests store less carbon^26^ than their less productive eastern counterparts^27^, and the same discrepancy is found between temperate and boreal forests^28^. In tropical Borneo, faster growing forests are found to contain less aboveground carbon density compared to slower growing sites simply due to differences in carbon residence time^29^. Although old trees reveal substantial annual biomass increments^22,25,30^, relatively little is known about the intraspecific tradeoffs between post-juvenile growth rates, plant height and stem diameter, as well as the lifespan of trees^31^. Are old, high-carbon-stock trees intrinsically slow growers^23^? Though, it has been argued that the tallest trees are also among the oldest^32^, it is not known whether this relates to lower growth rates earlier in life. If the size and/or age distribution of forest stands, together with the occurrence of disturbance events^33^, determines the extent of forest carbon stock, then the rate of forest carbon turnover (carbon residence time) is of paramount significance for estimating the long-term net CO_2_ capture from the Earth’s atmosphere^1–3,34,35^.

While the influence of climate change, nutrient availability and rising CO_2_ concentrations on tree growth can be examined experimentally under controlled conditions^34,36^, their influence on the lifespan of trees cannot. This is where dendroecology can provide unique insights into extra-tropical, inter-annual tree growth variability at centennial to millennial time-scales^37,38^. However, dendrochronological candidate collections must fulfill numerous criteria that are commonly not addressed in traditional dendroclimatological/ecological tree-ring studies^39,40^. To begin with, a large sample size of several hundreds to thousands of recent (living) and relict (dead) tree stems cross-sections, ideally consisting largely of disc samples that include the innermost ring, or core samples with reliable pith-offset estimates, are needed in order to provide sufficient statistical confidence for precise age and growth rate determinations. In addition, the species- and site-specific inventories of annual tree-ring width measurements, preferably from stem discs, rather than increment cores, must be characterized by a homogeneous distribution of the constituent series’ start and end dates over past centuries to represent pre-industrial climate conditions. The samples also need to contain a wide range of individual tree ages and growth levels. Moreover, the appropriate datasets should represent trees that grow in open environments, where year-to-year and longer-term ring width variations are largely constrained by growing-season temperatures, rather than between-tree competition. Finally, the collection sites should be free of silvicultural treatments, and minimally affected by natural disturbances^33^, such as cyclic insect defoliations and/or stochastic forest fires.

This study provides a conceptual framework to examine the growth-lifespan tradeoff in two conifer species and uses tree-ring width measurements from living and relict tree stem cross-sections to address the question: To what extent does the growth rate and lifespan of trees, within given species population, co-vary?

## Results

### Conceptual tree growth changes under global warming

**Fig. 1:**
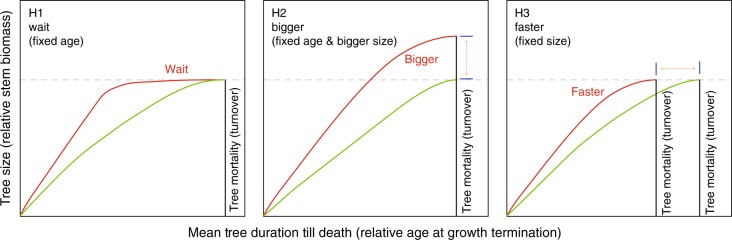
Conceptual diagram of tree growth under global warming. Three alternative hypotheses of how the relationship between the longevity (age) and growth rate (size) of trees may change under predicted global warming (red lines). H1 shows faster initial growth rates that are, however, not sustained until tree death (fixed-age hypothesis). H2 shows faster growth rates throughout the entire lifetime that is, however, not affected (bigger hypothesis). H3 shows faster growth rates together with a shorter lifespan (fixed-size hypothesis)

To meet the aforementioned criteria, and provide a dendroecological perspective on the relationship between plant lifespan and radial stem growth, we use annually resolved and absolutely dated tree-ring information from 1768 conifers that lived during the past two millennia in the Spanish Pyrenees and the Russian Altai. Due to the sites’ remote locations, we assume there are no direct anthropogenic disturbances affecting tree growth rates (as opposed to possible indirect affects vis-à-vis climate change). We consider the following three categorical hypotheses (Fig. 1), and acknowledge that reality maybe somewhere in between. H1 is the fixed-age hypothesis that states; if mean maximum tree size is reached early (growth has been accelerated for whatever reason), trees will wait to die until they reach a certain age. H2 is the bigger hypothesis that states; faster growing trees will become bigger (taller and greater stem diameter) within a given lifespan, and will die at a species-specific age. H3 is the fixed-size hypothesis that states; when trees grow faster, they will die once they reach a certain size, and that timing will determine the turnover rate. Under H1 and H2 tree growth patterns translate into increasing landscape-wide carbon stocks (sequestration), while the pattern predicted by H3 does not. An extension of H3 could include the possibility of the carbon stock being reduced by virtue of all intermediate responses between H2 and the accelerated turnover hypothesis. Our tests explore the importance of size versus age control on tree lifespan (H2 versus H3). Since neither the fatal consequences of great age or size can be expected to possess sharp thresholds, a large sample size is required to statistically describe tree lifespan (forest demography). Finally, we concede it is impossible to define explicitly the actual causes of tree mortality, because individuals may die due to just age or even age-related susceptibility to disturbance factors and/or post-disturbance pathogens.

### Grow fast—die young

**Fig. 2:**
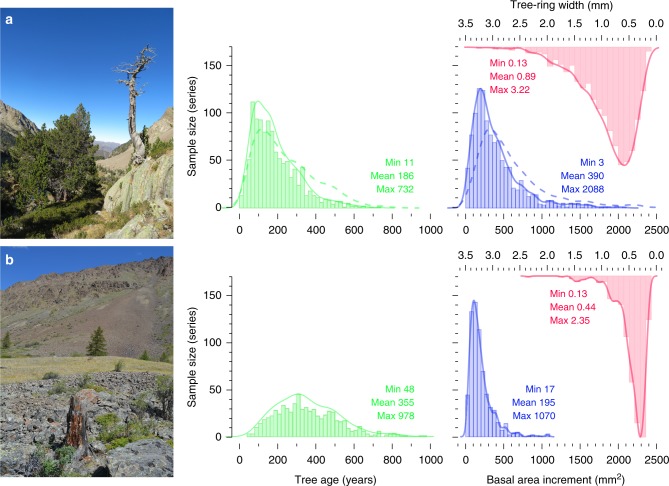
Tree growth characteristics. **a** Spanish Pyrenees and **b** Russian Altai where 1108 and 660 stem disc and increment core samples were collected from living (602 and 147) and relict (506 and 513) tree stems of Mountain pines (*Pinus uncinata* Ramond ex DC.) and Siberian larch (*Larix sibirica* Ledeb.), respectively. Histograms show the distribution of mean tree age (green), as well as mean annual tree-ring width and basal area increment (red and blue). Dashed lines refer the contribution made when considering pith-offset estimates (the number of missing rings between the innermost annual increment and the pith). Colored numbers show the minimum, mean and maximum (min, mean, max) values of tree age, tree-ring width and basal area increment (green, red, blue). Source data are provided as Source Data files

By analyzing 1108 tree-ring width and basal area increment series from 602 living and 506 dead Mountain pines (*Pinus uncinata* Ramond ex DC.) in the Spanish Pyrenees^41,42^, and 660 comparable samples (147 living and 513 relict tree stems) of Siberian larch (*Larix sibirica* Ledeb.) from the Russian Altai^43^ (Fig. 2), we reconstruct the total lifespan and juvenile growth rates of trees that were growing during both industrial and pre-industrial climate conditions (see Methods). All trees grew under undisturbed and unmanaged, summer temperature limited, high-elevation, climax forests with wide tree spacing in the Pyrenees and the Altai (see Methods). Since the lifespans of these trees from Europe and inner Eurasia are fairly evenly distributed over the past 1000 and 2000 years, respectively (Supplementary Fig. 1), the timing of each tree’s juvenile growth period occurred during different periods of natural climate variability and well before the recent warming. Most samples from the Pyrenees contain between ~100 and 200 growth rings (Fig. 2a), with mean ring widths between ~0.5 and 1.0 mm, which translates into a mean annual basal area increment of ~200–500 mm^2^. The much flatter age distribution of the Altai samples reveals the trees there are generally older and have slightly smaller annual increments (Fig. 2b). The Altai mean tree age, ring width and basal area are 355 years, 0.44 mm and 195 mm^2^, respectively.

**Fig. 3:**
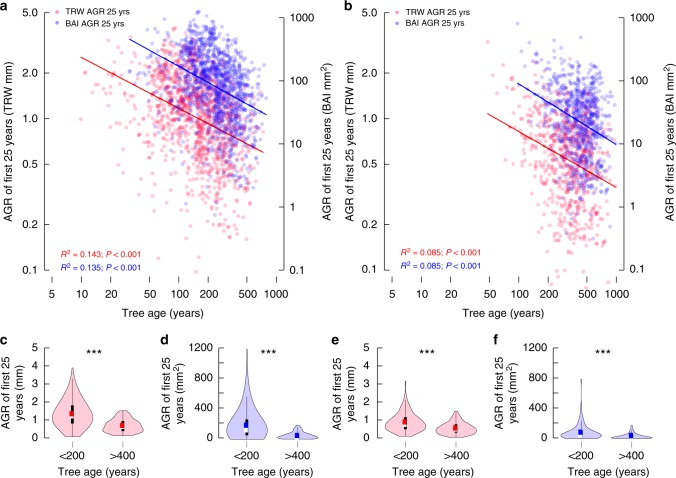
Tree growth and lifespan. Scatter plots of annual tree-ring width (TRW; red symbols and lines) and basal area increment (BAI; blue symbols and lines) averaged over the first 25 years of juvenile growth (AGR 25 yrs) in all living and relict tree stems from the Spanish Pyrenees (**a**) and Russian Altai (**b**). Each point represents one tree. Solid lines are linear models on log-transformed data, showing the tradeoff between tree growth and lifespan (productivity and longevity). Axes are log-scaled. Violin plots of annual tree-ring width (red) and basal area increment (blue) averaged over the first 25 years of juvenile growth in trees aged ≤ 200 and ≥ 400 years from the Pyrenees (**c**, **d**) and Altai (**e**, **f**). Violin plots show the full distribution of data, with white dots referring to the median, and colored dots describing mean tree-ring width (red) and basal area increment (blue). The extent of the black lines in the violins represents the interquartile ranges, and the light bars show the 95% confidence intervals. Asterisks indicate significant differences between the two age classes (Mann–Whitney U Test; ***p *<* 0.001). Source data are provided as Source Data files

The average ring width and basal area increment in the first 25 years of juvenile growth in the 1108 Pyrenees pine samples shows a clearly negative relationship with total tree lifespan (Fig. 3a). Old ages are reached only if juvenile growth is slow. Though less distinct a similar, and statistically significant, relationship can be seen in the 660 Altai larch samples (Fig. 3b). While the younger trees exhibit a wide range of growth rates, it is evident from both datasets that low juvenile growth rates are indeed required to reach a great tree age. Considering different periods of juvenile tree growth between 25 and 75 years, does not change this finding (Supplementary Table 1). Moreover, the association between increased juvenile stem growth and reduced total tree age remains statistically significant when calculated separately for the living and relict trees in both regions (Supplementary Table 1 and Supplementary Figs 2-3).

## Discussion

Based on the evidence of 749 living and 1019 relict tree-ring measurement series, representing species-specific conifer ring widths over the past 1000 and 2000 years at undisturbed high-elevation sites in the Spanish Pyrenees and Russian Altai, this study suggests that accelerated tree growth (past, present and future) is unlikely to translate into enhanced carbon sequestration, thereby mitigating the global greenhouse effect. The data illustrate that increased biomass productivity leads to reduced tree longevity (Fig. 1). A faster turnover of individual trees implies a shorter carbon residence time from stand to biome scales^2^ (in line with H3).

By accepting H3 and rejecting H1 and H2 (Fig. 1), we see no evidence for a shift in demography toward higher ages, and thus a greater carbon stock, when trees grow faster. Our data do support a size, rather than an age, control of tree lifespan and thus stand-level turnover, with recorded tree age being a consequence, rather than a cause, of death. Could faster growing trees break the size limit and get larger? This would require anatomical adjustments typically associated with maximum tree size that provide resilience in the face of the many physical disturbance vectors that damage trees^44^. Record tree heights found in both angiosperms (*Eucalyptus regnans* F.Muell.; mountain ash) and gymnosperms (*Sequoia sempervirens* (D.Don) Endl.; coastal redwood), are believed to relate to apical turgor maintenance^31,45^ irrespective of whether trees possess vessels or just tracheid cells, and explains why such giants are confined to humid areas^46^.

Given the data available, our findings are restricted to two conifer species of upper montane forests and the treeline ecotones, and therefore do not contribute to answering the question of how drought stress under predicted climate change will affect the functioning, productivity and carbon stocking of forest ecosystems at lower elevations^47–49^. Future estimates of the amount of stored carbon in arid environments are particularly challenging, as there is a thin line between drought-induced reductions of metabolic activity, which would extend the trees’ lifespan in line with our findings and facilitate long-term carbon storage, versus widespread forest dieback that would convert a carbon sink into a source, similar to what happens after large bark-beetle outbreaks and wildfires^50^. Any model-based predictions of how forest regrowth across different parts of the Earth’s landmass will affect global carbon dynamics are, however, associated with great uncertainties in the lights of demography^51^ and mortality^52^.

Although site-specific and species-specific, our results warn against scaling from growth rates to carbon stocking without accounting for tree lifespan and stand turnover (shifts in demography)^35^. The data presented here suggest that faster growth does not permit one to infer levels of carbon sequestration at the landscape scale. Such an inference would require responses closer to H1 and H2, which we did not find. Our data rather suggest that accelerated growth is associated with faster ontogeny, as was demonstrated by plantation trees exposed to elevated CO_2_^53^, and a higher likelihood of tree death as a function of tree size. The idea that global warming, artificial nitrogen deposition, or atmospheric CO_2_ enrichment will rise carbon stocks in forests, the size control of turnover hypothesis (extension of H3) must be rejected.

## Methods

### Tree-ring sampling

During several field campaigns since 2004, disc and core samples from 1108 living and dead Mountain pine (*Pinus uncinata* Ramond ex DC.) trees were collected at two upper treeline sites in the most northern part of the Aigüestortes i Estany de Sant Maurici National Park in the central Spanish Pyrenees^41,42^. This region is characterized by undisturbed, open ecotone habitats between around 2300 and 2600 m asl.

Over the past decade, stem discs and a few increment cores from 660 living and dead Siberian larch (*Larix sibirica* Ledeb.) trees were collected at five upper treeline sites across the Russian Altai-Sayan Mountains^43^. This region also is characterized by undisturbed, open forests around 2000–2400 m asl. High-elevation tree growth in both regions is predominantly controlled by summer temperature conditions. Individual trees at all sites can reach ages of up to 1000 years. The abundance of dead wood in the Altai and Pyrenees is indicative of remote locations with little to no modification by humans, or disturbance due to grazing by wild or domestic animals.

### Tree-ring analyses

All disc and core samples were air-dried and polished with sand paper of progressively finer grain size down to 800 grit. Tree-ring width (TRW) was measured at a resolution of 0.001 mm using LINTAB measuring systems, and cross-dated via TSAP-win and PAST4 software. All dating was verified with COFECHA (Version 6.02 P). The germination year (birth) of each tree was defined by the calendar date of its pith. In those cases where samples had no pith, pith-offset estimates were calculated, by fitting a geometric pith locator to the innermost rings and converting this distance into the number of missing rings. The dated TRW measurements were transformed into basal area increments (BAI) to account for the geometric constraints of adding incremental growth to an ever-increasing surface area.

Linear functions fitted to the log-transformed data of the first 25, 50, or 75 years of juvenile tree growth in all 1108 and 660 individual series of TRW and BAI from the Pyrenees and Altai, and plotted against total tree age, describe the overall tradeoff between the productivity (growth) and longevity (lifespan) of trees. Mean TRW and BAI of the juvenile tree growth of all samples ≤ 200 and ≥ 400 years further emphasize the tendency of younger trees to grow faster and older trees to grow slower. Finally, we performed the same analysis on the 506 and 513 relict (602 and 147 living) trees from the Pyrenees and Altai to test for the temporal stability in our results.

## Supplementary information

Supplementary Information

Source Data

## Data Availability

All source data underlying this study are provided as two separate Source Data files, for the Spanish Pyrenees (SourceDataPyrenees.txt) and the Russian Altai (SourceDataAltai.txt). All calculations were performed with the open access software R.
