# Author Correction: Limited capacity of tree growth to mitigate the global greenhouse effect under predicted warming

**DOI:** 10.1038/s41467-019-10731-x

**Published:** 2019-06-17

**Authors:** Ulf Büntgen, Paul J. Krusic, Alma Piermattei, David A. Coomes, Jan Esper, Vladimir S. Myglan, Alexander V. Kirdyanov, J. Julio Camarero, Alan Crivellaro, Christian Körner

**Affiliations:** 1 Department of Geography, University of Cambridge, Cambridge, CB2 3EN UK; 2 Swiss Federal Research Institute (WSL), 8903 Birmensdorf, Switzerland; 3 Global Change Research Centre and Masaryk University, 613 00 Brno, Czech Republic; 4 Department of Physical Geography, Stockholm University, 10691 Stockholm, Sweden; 5 Department of Plant Sciences, University of Cambridge, Cambridge, CB2 3EA UK; 6 Departmemt of Geography, Johannes Gutenberg University, 55099 Mainz, Germany; 7 Institute of Humanities, Siberian Federal University, 660041 Krasnoyarsk, Russia; 8 Sukachev Institute of Forest SB RAS, 660036 Krasnoyarsk, Russia; 9 Institute of Ecology and Geography, Siberian Federal University, 660041 Krasnoyarsk, Russia; 10 Instituto Pirenaico de Ecología (IPE-CSIC), 50059 Zaragoza, Spain; 11 Institute of Botany, University of Basel, 4056 Basel, Switzerland

**Keywords:** Ecology, Climate-change ecology, Ecosystem ecology, Ecosystem services, Forest ecology

Correction to: *Nature Communications* 10.1038/s41467-019-10174-4, published online 15 May 2019.

The original HTML version of this Article was updated after publication to add links to the Source Data.

