# Supplementary material for: Limited capacity of tree growth to mitigate the global greenhouse effect under predicted warming
Source: Nat Commun. 2019 May 15;10:2171. doi: 10.1038/s41467-019-10174-4 (PMC6520339; doi:10.1038/s41467-019-10174-4)
Supplement: Supplementary file 1 — Supplementary Information [file 41467_2019_10174_MOESM1_ESM.pdf]

Supporting Information

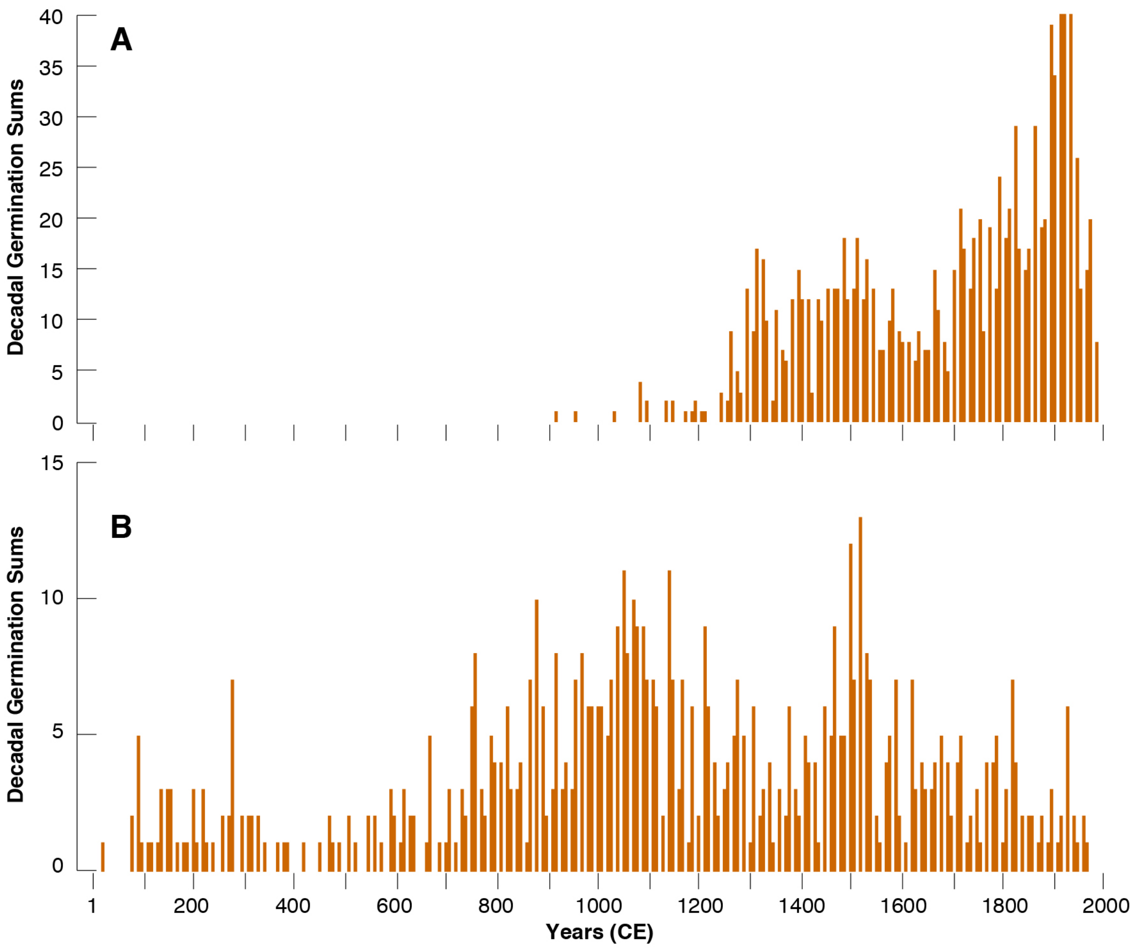

Supplementary Figure 1. Decadal-sums of all germination dates associated with the 1108 pine (**A**) and 660 larch (**B**) trees from the Spanish Pyrenees and the Russian Altai, respectively. Source data are provided as Source Data files.

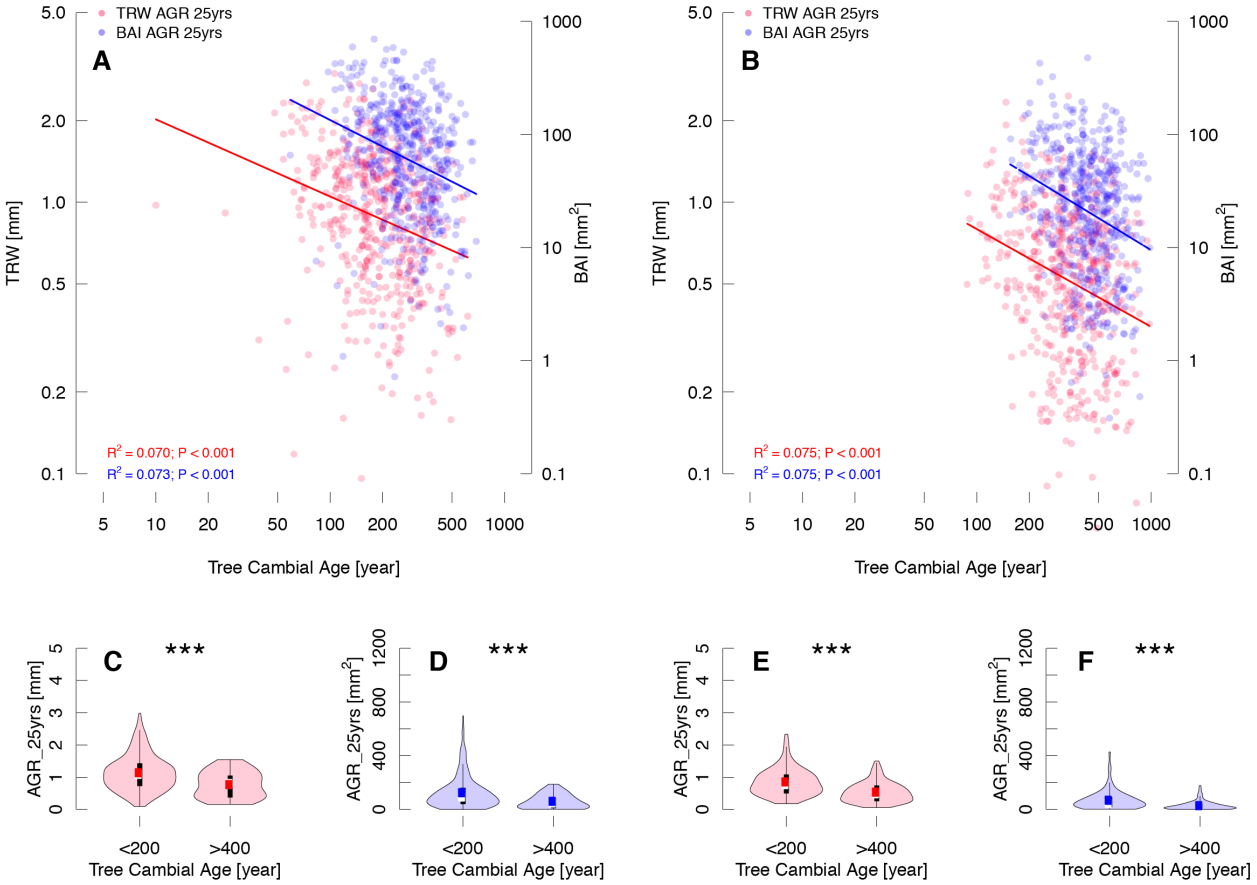

Supplementary Figure 2. Tree growth rates and lifespan of the relict trees that were growing under natural, pre-industrial climate conditions. Scatter plots of annual tree-ring width (TRW; red symbols and lines) and basal area increment (BAI; blue symbols and lines) averaged over the first 25 years of juvenile growth (AGR 25yrs) of 506 relict tree stems from the Spanish Pyrenees (**A**) and 513 relict tree stems from the Russian Altai (**B**). Each data point represents one tree. Solid lines are linear models on log-transformed data, showing the tradeoff between tree growth and lifespan (productivity and longevity). All axes are log-scaled. Violin plots of annual tree-ring width (red) and basal area increment (blue) averaged over the first 25 years of juvenile growth in trees aged ≤200 and ≥400 years from the Spanish Pyrenees (**C, D**) and Russian Altai (**E, F**). Violin plots show the full distribution of the data, with the white dots showing the median, and the

colored dots showing the mean for tree-ring width (red) and basal area increment (blue). The extent of the black lines in the violins represents interquartile ranges, and the light bars show the 95% confidence intervals. Asterisks indicate significant differences between the two age classes (Mann-Whitney U Test; *** p <0.001). Source data are provided as Source Data files.

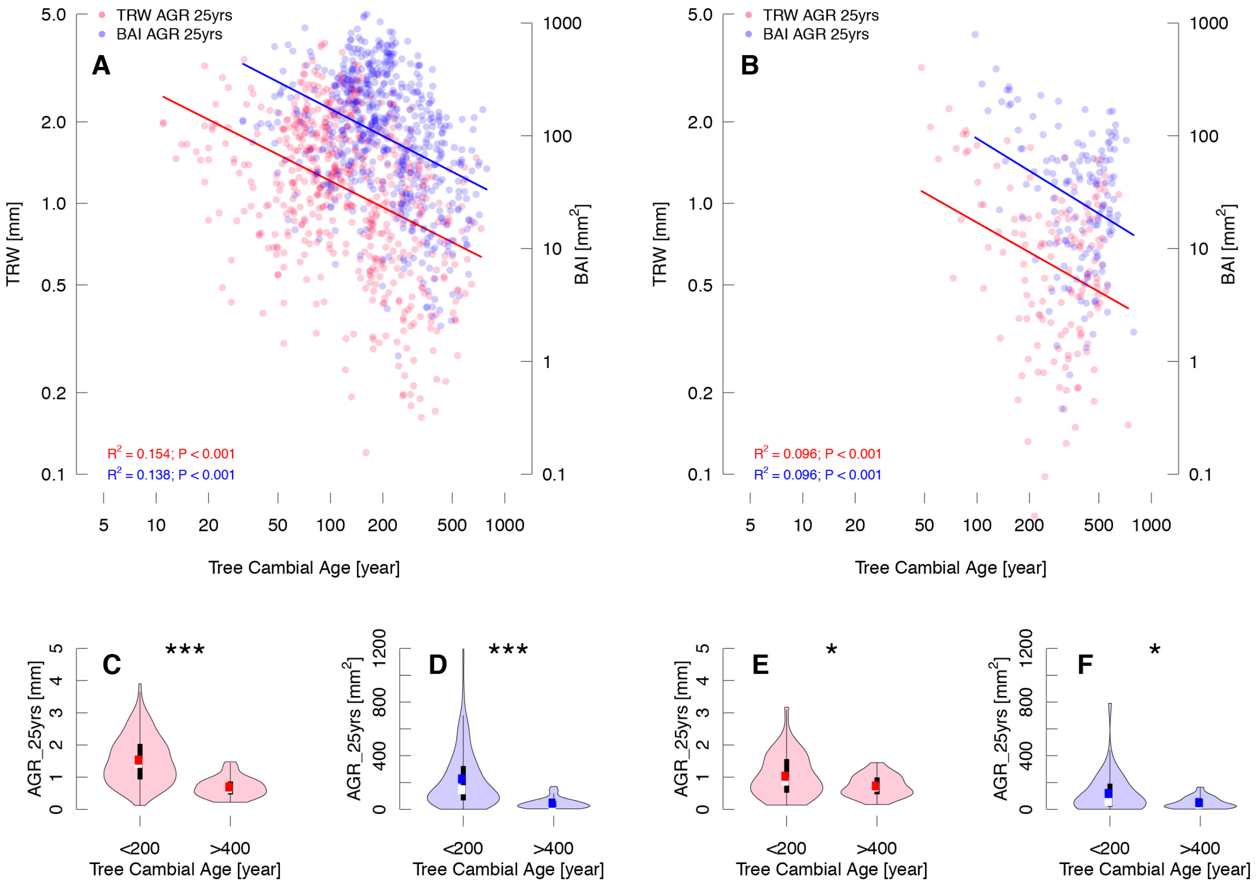

Supplementary Figure 3. Tree growth rates and lifespan of the living trees that are growing under anthropogenic, industrial climate conditions. Scatter plots of annual tree-ring width (TRW; red symbols and lines) and basal area increment (BAI; blue symbols and lines) averaged over the first 25 years of juvenile growth (AGR 25yrs) of 602 relict tree stems from the Spanish Pyrenees (**A**) and 147 relict tree stems from the Russian

Altai (**B**). Each data point represents one tree. Solid lines are linear models on log-transformed data, showing the tradeoff between tree growth and lifespan (productivity and longevity). All axes are log-scaled. Violin plots of annual tree-ring width (red) and basal area increment (blue) averaged over the first 25 years of juvenile growth in trees aged ≤200 and ≥400 years from the Spanish Pyrenees (**C, D**) and Russian Altai (**E, F**). Violin plots show the full distribution of the data, with the white dots showing the median, and the colored dots showing the mean for tree-ring width (red) and basal area increment (blue). The extent of the black lines in the violins represents interquartile ranges, and the light bars show the 95% confidence intervals. Asterisks indicate significant differences between the two age classes (Mann-Whitney U Test; *** p <0.001, * p <0.05). Source data are provided as Source Data files.

Supplementary Table 1. Statistical test of tree-ring width (TRW) and basal area inclement (BAI) from the living and relict tree stems in the Spanish Pyrenees and Russian Altai. The slope, R^2 and p-values are provided for each linear model on log-transformed data of the Average Growth Rate (AGR) of the first 25, 50 and 75 years against total tree age. For both of the two separate age classes ≤200 and ≥400 years, the AGR, number of trees, mean, and standard deviation (s.d.) over the first 25, 50 and 75 years of growth is shown. Mean differences between age classes are tested with Mann-Whitney U Test (*** p <0.001, ** p <0.01, * p <0.05). Source data are provided as Source Data files.

			Linear model on log transformed data for Average Growth Rate (AGR) vs tree cambial age				Average Growth Rate (AGR)											
							AGR for trees aged ≤200 years			AGR for trees aged ≥400 years			25		50		75	
	Site	Status	AGR years	Slope (mm yr^{-1} or mm^2 yr^{-1})	R^2	p-value	No. trees	Mean (mm or mm^2)	s.d. (mm or mm^2)	No. trees	Mean (mm or mm^2)	s.d. (mm or mm^2)	≤ 200	≥ 400	≤ 200	≥ 400	≤ 200	≥ 400
Tree ring Width (TRW)	Pyrenees	All	25	-0.336	0.142	***	710	1.37	0.70	68	0.72	0.35	***					
			50	-0.318	0.154	***	710	1.27	0.60	68	0.68	0.30			***			
			75	-0.315	0.170	***	710	1.18	0.54	68	0.64	0.26					***	
		Living	25	-0.324	0.154	***	426	1.53	0.75	39	0.69	0.31	***					
			50	-0.312	0.170	***	426	1.39	0.63	39	0.64	0.25			***			
			75	-0.316	0.197	***	426	1.28	0.54	39	0.60	0.21					***	
		Relict	25	-0.284	0.070	***	284	1.14	0.56	29	0.78	0.40	***					
			50	-0.271	0.076	***	284	1.09	0.52	29	0.74	0.36			***			
			75	-0.260	0.078	***	284	10.2	0.49	29	0.70	0.30					***	
	Altai	All	25	-0.369	0.085	***	118	0.91	0.52	234	0.57	0.32	***					
			50	-0.395	0.117	***	118	0.88	0.45	234	0.53	0.28			***			
			75	-0.412	0.141	***	118	0.84	0.41	234	0.50	0.25					***	
		Living	25	-0.365	0.096	***	45	1.03	0.66	39	0.72	0.33	*					
			50	-0.384	0.127	***	45	0.97	0.56	39	0.65	0.28			*			
			75	-0.410	0.162	***	45	0.94	0.51	39	0.60	0.24					**	
		Relict	25	-0.360	0.075	***	79	0.85	0.42	189	0.54	0.31	***					
			50	-0.398	0.109	***	79	0.83	0.39	189	0.51	0.27			***			
			75	-0.413	0.130	***	79	0.80	0.34	189	0.48	0.25					***	
Basal Area Index (BAI)	Pyrenees	All	25	-0.654	0.135	***	709	186.09	186.12	68	51.41	46.38	***					
			50	-0.528	0.107	***	709	296.86	280.26	68	88.28	74.99			***			
			75	-0.414	0.075	***	709	352.80	322.01	68	111.89	85.81					***	
		Living	25	-0.611	0.138	***	426	225.31	210.05	39	45.08	39.99	***					
			50	-0.468	0.098	***	426	342.00	305.06	39	74.47	57.15			***			
			75	-0.350	0.062	***	426	390.48	336.19	39	94.52	66.52					***	
		Relict	25	-0.598	0.073	***	283	127.04	121.05	29	59.94	53.35	***					
			50	-0.557	0.076	***	283	228.92	221.91	29	106.84	91.66			***			
			75	-0.485	0.065	***	283	296.09	290.88	29	135.25	103.08					***	
	Altai	All	25	-0.738	0.085	***	118	86.34	106.35	234	34.01	36.38	***					
			50	-0.789	0.117	***	118	152.97	160.38	234	57.50	57.65			***			
			75	-0.813	0.138	***	118	200.62	186.65	234	74.79	74.70					***	
		Living	25	-0.730	0.096	***	39	117.89	149.10	45	49.39	41.90	*					
			50	-0.767	0.126	***	39	195.34	198.25	45	78.05	60.44			*			
			75	-0.784	0.152	***	39	248.06	220.93	45	96.80	69.30					**	
		Relict	25	-0.720	0.075	***	79	70.76	73.43	189	30.36	34.05	***					
			50	-0.795	0.109	***	79	132.06	134.52	189	52.61	56.03			***			
